# Computational fluid dynamics comparison of the upper airway velocity, pressure, and resistance in cats using an endotracheal tube or a supraglottic airway device

**DOI:** 10.3389/fvets.2023.1183223

**Published:** 2023-09-25

**Authors:** Carla Zamora-Perarnau, Mauro Malvè, Rocío Fernández-Parra

**Affiliations:** ^1^Doctoral School, Catholic University of Valencia San Vicente Mártir, Valencia, Spain; ^2^Department of Small Animal Medicine and Surgery, Faculty of Veterinary Medicine, Catholic University of Valencia San Vicente Mártir, Valencia, Spain; ^3^Veterinary Referral Hospital UCV, Catholic University of Valencia San Vicente Mártir, Valencia, Spain; ^4^Department of Engineering, Public University of Navarre (UPNA), Pamplona, Spain; ^5^Biomedical Research Networking Center in Bioengineering, Biomaterials, and Nanomedicine (CIBER-BBN), Madrid, Spain

**Keywords:** *in silico*, airway management, v-gel^®^, feline, general anesthesia, ETT, flow

## Abstract

**Intoduction:**

In veterinary medicine, airway management of cats under general anesthesia is performed with an endotracheal tube (ETT) or supraglottic airway device (SGAD). This study aims to describe the use of computational fluid dynamics (CFD) to assess the velocities, pressures, and resistances of cats with ETT or SGAD.

**Methods:**

A geometrical reconstruction model of the device, trachea, and lobar bronchi was carried out from computed tomography (CT) scans that include the head, neck, and thorax. Twenty CT scans of cats under general anesthesia using ETT (*n* = 10) and SGAD (*n* = 10) were modeled and analyzed. An inspiratory flow of 2.4 L/min was imposed in each model and velocity (m/s), general and regional pressures (cmH_2_O) were computed. General resistance (cmH_2_O/L/min) was calculated using differential pressure differences between the device inlet and lobar bronchi. Additionally, regional resistances were calculated at the device’s connection with the breathing circuit (region A), at the glottis area for the SGAD, and the area of the ETT exit (bevel) (region B) and the device itself (region C).

**Results:**

Recirculatory flow and high velocities were found at the ETT’s bevel and at the glottis level in the SGAD group. The pressure gradient (Δp) was more enhanced in the ETT cases compared with the SGAD cases, where the pressure change was drastic. In region A, the Δp was higher in the ETT group, while in regions B and C, it was higher in the SGAD group. The general resistance was not statistically significant between groups (*p* = 0.48). Higher resistances were found at the region A (*p* = <0.001) in the ETT group. In contrast, the resistance was higher in the SGAD cases at the region B (*p* = 0.001).

**Discussion:**

Overall, the provided CT-based CFD analysis demonstrated regional changes in airway pressure and resistance between ETT and SGAD during anesthetic flow conditions. Correct selection of the airway device size is recommended to avoid upper airway obstruction or changes in flow parameters.

## 1. Introduction

Airway management in veterinary medicine is vital when patients are unable to maintain airway patency autonomously, especially in emergencies and during an anesthesia. Orotracheal intubation with an endotracheal tube (ETT) maintains airway patency, allows positive pressure ventilation, ss aspiration of any material entering the oropharynx, and allows delivery of inhaled anesthetic gasses and oxygen to the patient (1). In cats, however, laryngeal spasm (2), soft tissue swelling (3), tracheal ruptures (1, 3, 4), sublaryngeal tracheal injury and ulceration (5) or trauma to arytenoid cartilages (1, 3) have been reported. Alternatively, a laryngeal mask or supraglottic airway device (SGAD) has been developed for airway management during anesthesia. These devices seal the upper airways above the rima glottidis and are associated with an increased risk of gastro-esophageal reflux and possible aspiration in human (2) and veterinary patients (6). They were first developed for humans (7) and then for rabbits and cats (6, 8, 9). The SGAD used in cats is positioned above the rima glottidis, seal the esophageal entrance, and an inflatable dorsal adjuster that can increase seal pressure allowing spontaneous or mechanical ventilation (9). Insertion problems have been described, including multiple attempts to correct placement, dislodgement, coughing during insertion, causing upper airway obstruction, stimulus for regurgitation and vomiting (3, 6) and fresh gas flow leakages during mechanical ventilation (3).

Computational fluid dynamics (CFD) is a non-invasive *in silico* technique that uses numerical algorithms to solve the governing equations of fluid dynamics and characterize the flow in numerous clinical situations (10, 11). This technique uses images from computed tomography (CT) or magnetic resonance imaging (MRI) scans of patients to create reconstructions of specific anatomical geometries. CFD is used in many fields like scientific research, clinical practice, industry or biomedical engineering. Normal and diseased human breathing and flow behavior have been largely studied in the last decades (10, 12). This is a relatively new technique in the veterinary sciences. It has been recently applied to the canine upper airways to compare upper airway pressure and resistance in brachycephalic, mesocephalic, and dolichocephalic dogs (13) and nasal flow resistance in English bulldogs (14). It has been used to compare respiratory function in brachycephalic patients (before and after surgery for the brachycephalic obstructive airway syndrome) (15, 16) and the evaluation of transport, distribution, and deposition of inhaled salbutamol particles in upper and lower airways in cats (15). Other studies focused on respiratory anatomy and respiratory physiology of animals, for example in bats (16), rabbits (17, 18), rats (19–21), pigs (22), mice (23), wild cats (24), dogs (25, 26), deer (27) and monkeys (19, 28) or for setting up animal models for human medicine (29, 30).

Understanding airway fluid dynamics is important for studying drug delivery, particle inhalation, airway disease, ventilation, and breath sound generation (22). The upper airways contribute to most flow resistance of the respiratory tract, and thus, abnormal anatomy of the upper airways can have consequences on the flow, like in brachycephalic syndrome (31). In general, the relationship among flow, pressure and resistance can be expressed through the analogy of the Ohm’s law as pressure difference or pressure gradient (Δp) = flow × resistance. Hence, resistance = pressure difference (cmH_2_0)/flow (L/min). Flow is defined as the quantity of a fluid passing through a specific location per unit time and it can develop different regimes, depending on several aspects related to the fluid properties, to the geometry and to the velocity (32). During the passage of the air across the glottis, a physiological Δp occurs, caused by anatomical changes in the larynx cross-section (33) which leads to increased flow velocity. When an individual inhales, the laminar flow becomes turbulent in the larynx and tends to be laminar in the lower airways (34). The flow regime (turbulent or laminar) is determined by the Reynolds number. When de Reynolds number is less than 2000, flow is predominantly laminar, and when it is greater than this number, turbulent flow dominates (32) (see Suplemmentary material – Supplementary Text: physics). When the tube wall can be considered rigid, the flow is governed by the Poiseuille’s Law that states the proportionality between the Δp and the flow *ϕ* times the resistance *R* (Δp ∝ *R ϕ*). The resistance is proportional to the dynamic viscosity of the fluid and the length of the tube while it is inversely proportional to the fourth power of the tube radius (see Suplemmentary material – Supplementary Text: physics). These variables can be computed in different clinical situations starting from CT or MRI images (33), using numerical algorithms to solve the Reynolds-averaged Navier–Stokes equations that describe the flow motion in different conditions with complex geometries (for furter details see Suplemmentary material – Supplementary Text: physics).

In our veterinary teaching hospital, we observed that cats under general anesthesia in which we used SGAD presented CT images with a partial or complete rima glottidis closure and esophageal aerophagy, during respiratory work-ups. As well, signs of airway obstruction were clinically observed with the capnogram (phase II slope upward with a blunted α angle in the capnograph) and, in some cases, reintubation was needed by our anesthesiolgist. These clinical situation led us to the aim of this study that was to use CFD for the investigation of the airway velocity, pressure between different regions and resistances in the upper airways of cats intubated with ETT compared to SGAD. We hypothesized that SGAD would cause more airway resistance and pressure compared with ETT, specifically at the glottis region, with more areas of recirculatory flow because of its morphology.

## 2. Materials and methods

### 2.1. Animal use and ethical approval

In this retrospective and comparative study, cats that underwent head, neck, and thorax CT scans as part of a diagnostic protocol at the Veterinary Referral Hospital UCV, Catholic University of Valencia San Vicente Mártir from 2018 to 2022 were enrolled for further analysis. All patients were part of the hospital’s clinical cases for which CT scans had to be performed as part of their diagnostic protocol. All enrolled cats required anesthesia for CT examination, and they were either intubated with an ETT or had their airway patency maintained with a SGAD (v-gel^®^ for cats sizes C1-C6, Docsinnovent Ltd., Hertfordshire, United Kingdom). Anesthesia protocol was adapted to each cat, and food (but no water) was withdrawn 12 h before the procedure. Information such as breed, sex, neutered status, age, weight, size of the device used in the procedure, and the presence or absence of clinical respiratory signs (coughing, sneezing, tachypnea), was also recorded. The presence of bronchial or tracheal intraluminal obstruction or collapse, incomplete CT scan studies, and/or any technical image acquisition issue, such as motion artifacts, were exclusion criteria. All procedures were conducted as part of standard veterinary clinical practice with the owner’s consent, and approval from the institutional ethical committee (CEEAUCV2101) was obtained. Twenty cats, either with an ETT (*n* = 10) or with a SGAD (*n* = 10), were included.

### 2.2. Image acquisition and analysis

All CT scans were performed using a multidetector 16-slice CT scanner (Siemens Somatom Scope, Munich, Germany) in helical scan mode. All cats were positioned in sternal recumbency, and scans of the head, neck, and thorax were performed (entire study or for regions acquired separately), including from the connection of the breathing circuit to the most cranial part of the liver. Computed tomography acquisition protocols and technique settings for CT scans included: 512 × 512 matrix, a pitch of 0.65 with scan thickness of 0.75–1.5 mm, 220 mAs, 130 kV, and a patient size adjusted display field of view. At the end of the CT examination, the cats were supervised until they recovered or remained anesthetized for further procedures.

Images were viewed using two DICOM (Digital Imaging and Communications in Medicine) viewers (Aycan Workstation PRO software v3.16.010 SW and Horos v4.0.0 RC5) with a lung (window width [WW] = 1,400 HU (Hounsfield units) and window level [WL] = −500 HU), soft tissue (WW = 120 HU; WL = 40 HU) and bone (WW = 1,500 HU; WL = 300 HU) reconstruction algorithms.

**Figure 1:**
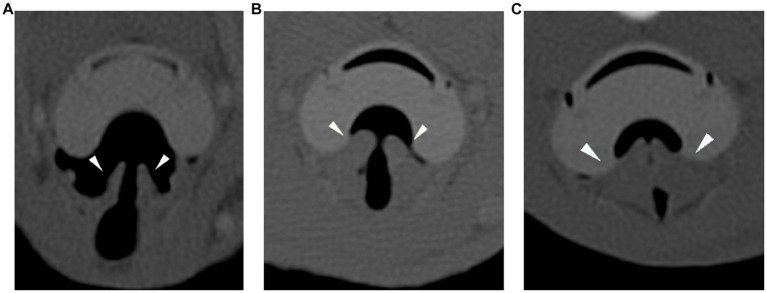
Transverse plane of the CT-scan in bone reconstruction algorithm for the three degrees of rima glottidis closure (arrowheads). Grade 0 = open glottis **(A)**, grade 1 = glottis partially closed **(B)**, and grade 2 = closed glottis **(C)**. CT: computed tomography.

In the CT images, the ETT bevel’s orientation to the tracheal wall was described in all cases as ventral, lateral, dorsal, or aligned to it. In the SGAD cases, a classification for rima glottidis closure based on 3 grades was proposed for this study (grade 0 = open glottis; grade 1 = glottis partially closed; grade 2 = closed glottis) (see Figure 1). In this article, we refer to rima glottidis as the opening between the vocal cords and as part of the glottis, which is the anatomical section itself.

### 2.3. Geometrical reconstruction and numerical discretization

**Figure 2:**
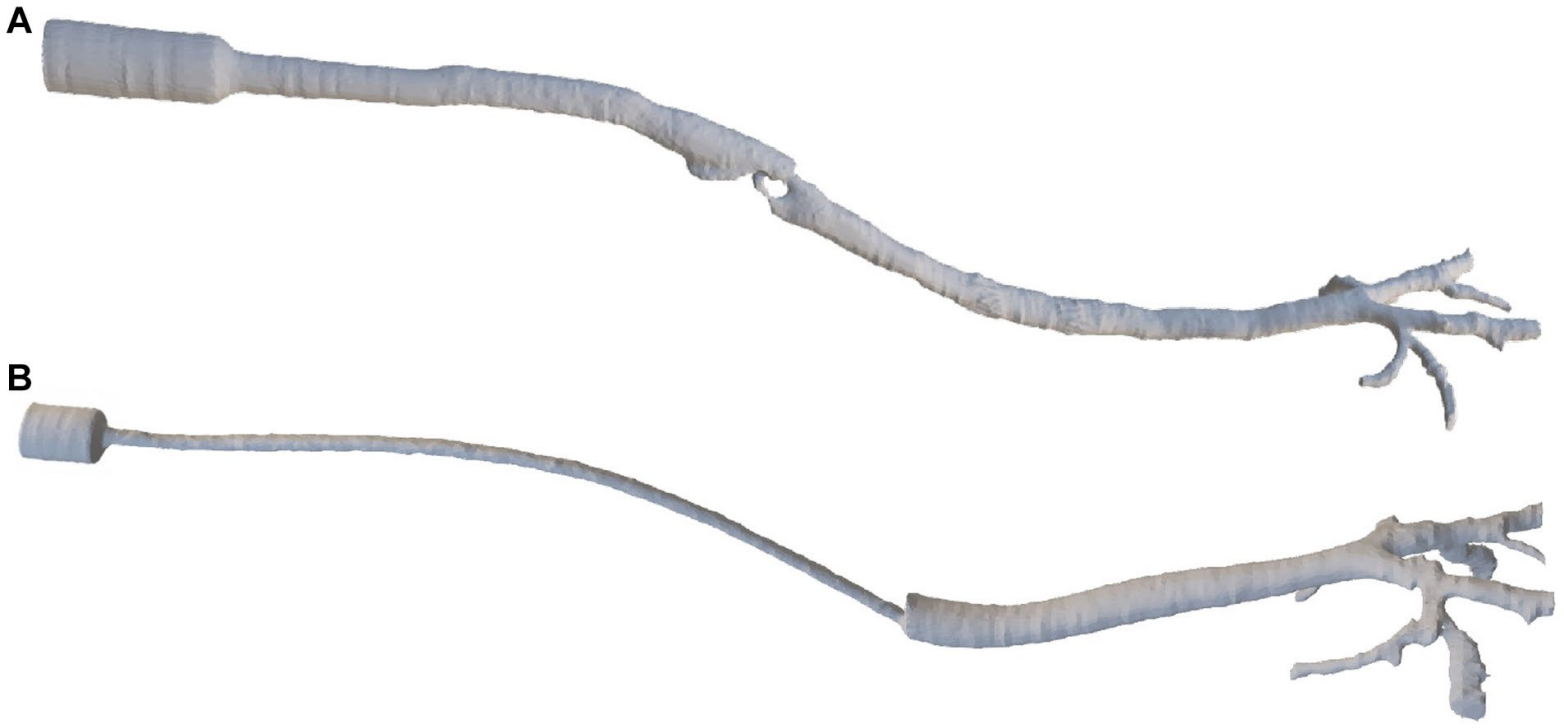
The final reconstruction of a SGAD (Case #5) **(A)** and an ETT (Case #4) **(B)** via image-based geometry reconstruction software (MIMICS, Materialise Software). ETT: endotracheal tube; SGAD: supraglottic airway device.

The DICOM files derived from the CT scans were imported into the image-based geometry reconstruction software (MIMICS, Materialise Software, Leuven, Belgium). Manual reconstruction of the device (ETT and SGAD), larynx in the case of SGAD, trachea, mainstem bronchi, and each lobar bronchus geometry were conducted for each cat (Figure 2). A stereolithography (STL) file was exported in each case.

**Figure 3:**
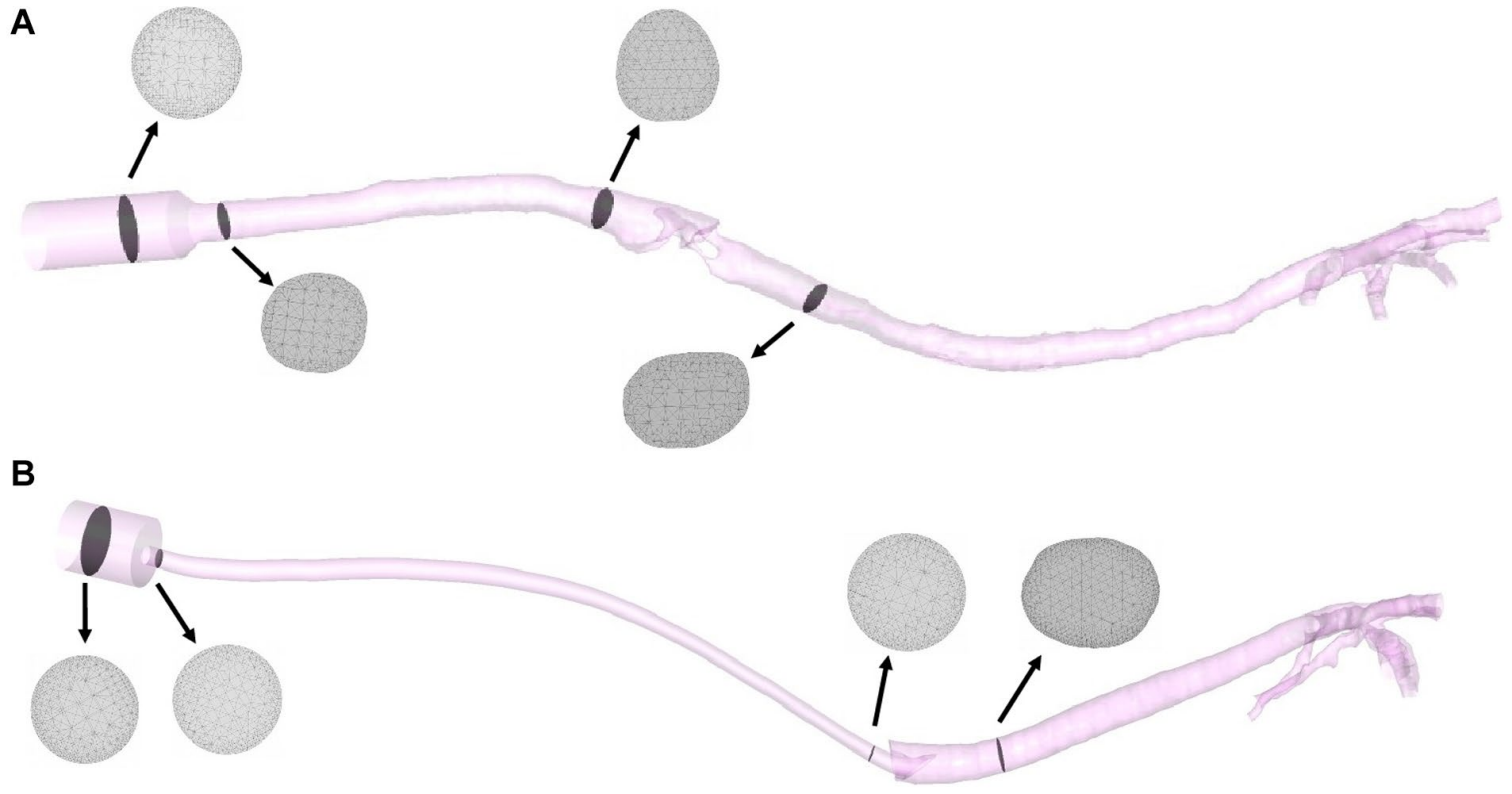
Final models of a SGAD (Case #5) **(A)** and an ETT (Case #4) **(B)** with four planes (gray) representing the computational grids. ETT: endotracheal tube; SGAD: supraglottic airway device.

STL files for the patient-specific models were imported to the commercial software package Rhinoceros (v. 5 SR14 32-bit, Robert McNeel and Associates, Seattle, WA, United States), and the ETT and SGAD geometries were added to the cat models. Inlet and outlet sections were created, and all the surfaces building the 3D volumes were exported again as STL files. These files were imported in the software package Ansys IcemCFD (v. 22.2, Ansys Inc., Canonsburg, PA, United States), where the volume of each model was subdivided into tetrahedral elements. At this stage, the minimum size of all tetrahedral edge lengths was specified to control the number of elements. Near-wall regions required a denser mesh with more elements to increase the accuracy of the geometry of the small airways and the laryngeal region in the SGAD cases. The element minimum edge length required varied depending on the size and morphology of the airway region or device, generating slightly different computational grids for the spatial resolution (see Figure 3). Before the final mesh creation, a mesh-independent study was performed to ensure adequate grid sizes. The study, conducted on the airway velocity profiles inside the trachea and the main bronchi with both devices, demonstrated that for both devices, computational grids over 2 million provided similar results in terms of velocity profiles (relative difference between profiles less than 2%). Considering that the Δp, as widely demonstrated in the literature, are relatively unaffected by the element size (25) we concluded that grids ranging from 2 to 5 million elements (Suplemmentary material – Supplementary Table 1), depending on the model geometry, were sufficient for describing the flows within the patient-specific models.

### 2.4. CFD analysis

The numerical grids from the 20 cats were imported into the commercial software package ANSYS CFX, v.22.2 (Ansys Inc., Canonsburg, PA, United States). This software uses numerical algorithms to solve the Reynolds-averaged Navier–Stokes equations that describe the flow motion in different conditions within the geometrical grids. In particular, the Ansys CFX software adopts the finite volume method. The software manual provides the exact mathematical formulations and the solving algorithms used by Ansys CFX (Ansys, 2022).

**Figure 4:**
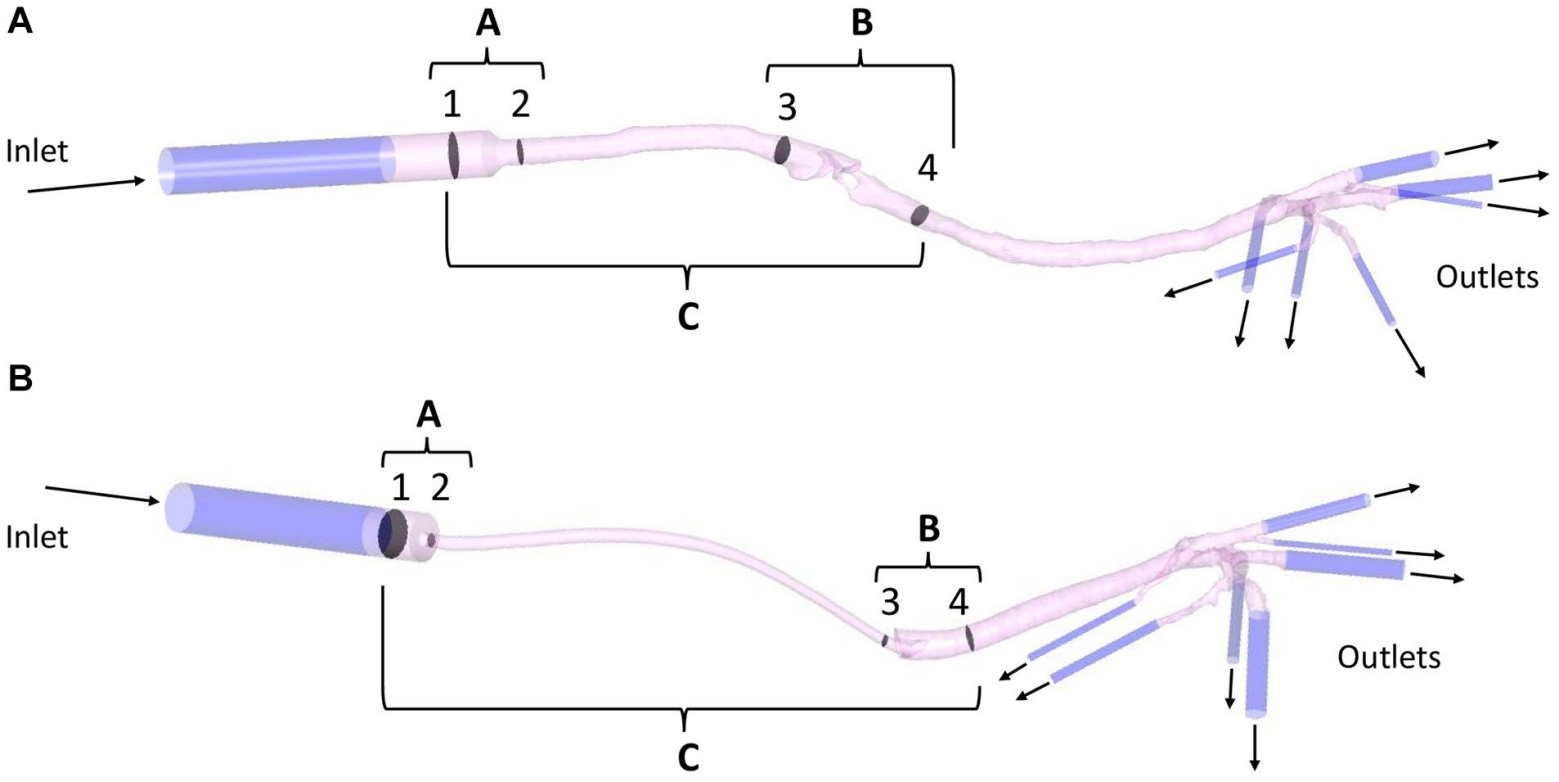
Example of a SGAD (Case #5) **(A)** and an ETT (Case #4) **(B)** model with regions A, B, and C formed by planes 1–2, 3–4, and 1–4, respectively. Regional pressure, pressure gradient, and resistance were calculated in three sections, between planes 1–2 (region A), 3–4 (region B), and 1–4 (region C). Representation of inlet and outlet extensions (dark blue) added to each model (pink). ETT: endotracheal tube; SGAD: supraglottic airway device.

For boundary conditions, a peak inspiratory flow of 2.4 L/min was imposed at the inlet of each model and, at the outlets, a zero-pressure condition was given. The flow value was obtained using the peak value of different spirometry curves (Carescape Monitor B650, General Electric, Boston, MA, United States with the spirometer module E-sCAiOV and pitot sensor Pedi-Lite) in cats under general anesthesia. To dampen the effect of the boundary conditions, 5-diameter inlet and outlet extensions were added to the model (see Figure 4). The simulation used the following fluid properties: air density 1.225 kg/m^3^, viscosity 1.83·10^−5^ kg/(m·s) and 25°C. The flow was considered steady (constant inspiratory peak flow) (35, 36) and turbulent (Reynolds number > 2,000). The *k-ω* SST (Shear Stress Transport) turbulence model was used with an initial turbulence intensity value of 5%. The numerical discretization and computational cost of each mesh are summarized in the Suplemmentary material (see Supplementary Table 1).

### 2.5. Resistance evaluation

General resistance was calculated using the pressure gradient of the whole model with the following equation:


$$\mathrm{Resistance}\;\left(\mathrm{cm}H_{2}O/L/\min\right)=\frac{\Delta p\;\left(\mathrm{cm}H_{2}O\right)}{\mathrm{flow}\;\left(L/\min\right)}$$

where Δp is the pressure gradient between maximum and minimum general pressure. The flow (2.4 L/min) was the same in all cases.

Regional resistance evaluation was determined using the simulation results. The flow and pressure maps were evaluated by looking for areas of flow recirculation and/or abrupt Δp, respectively. The higher flow recirculation and Δp was localized at the glottis area in the SGAD group. At the ETT group, two areas were observed, at the ETT connection to the breathing circuit and where bevel contacts the tracheal wall. The model was divided into sections for further analysis. Section 1 was the proximal connection of the device to the breathing circuit, section 2 the proximal region where the device’s diameter decreases. Sections 3 and 4 are, 2 cm cranial and caudal to the glottis in the SGAD and 1 cm cranial and caudal to the ETT bevel, respectively. Then the regional Δp was computed at three regions (A, B, and C) of each anatomical model with the previously mentioned equation. The Δp at the device’s entrance at sections 1 and 2 (region A), at sections 3 and 4 (region B), and between sections 1 and 4 (region C, the entire device) were computed (see Figure 4).

### 2.6. Statistical analysis

Sample size calculations indicated that 10 cats per group was sufficient to detect a decrease in resistance of 25%, mean of 38.7 cmH_2_O/L/s, and standard deviation (± SD) of 7.8 (pilot study (37)). A statistical power of 0.8 and α value of 0.05 (two-tailed analysis for paired data was input into the online sample size calculator https://clincalc.com/stats/samplesize.aspx). The measured data were analyzed using the IBM^®^ SPSS^®^ Statistics software version 27 (Chicago, IL, United States). A *Shapiro–Wilk* test was used to test the normal distribution of Δp values (general and regional), resistance (general and regional), and velocities. The results are expressed as medians (maximum and minimum range). *Mann–Whitney U* tests compared general and regional Δp values, general and regional resistance, and velocities between groups (ETT/SGAD). Values of *p* < 0.05 were considered statistically significant.

## 3. Results

A total of 40 cat CT scans were obtained and reviewed. Thirteen SGAD and 16 ETT cases were selected and 9 cats were excluded. One SGAD case was excluded because of bronchial collapse, another because of a tracheal mass, and the other case did not have the airways wholly included in the CT scan. In the ETT group, two cases were excluded because the presence of soft tissue in the bronchi, and one was incorrectly positioned in the CT scan making difficult correct reconstruction. Three ETT cases did not have the airways wholly included in the CT scan. Twenty CT studies, either with an ETT (*n* = 10) or a SGAD (*n* = 10), were finally included.

The ETT group age range was between 3 months and 19 years with a mean age of 7 years, and in the SGAD group, the age range was between 1 to 8 years with a mean age of 4 years. The breeds included Turkish mohair, Siamese (two), Persian, and Domestic Shorthair (16). Body weight, ETT internal diameter (ID) in mm, the SGAD sizes, the orientation of the bevel, the slice thickness of the CT and the glottic closure grade in the SGAD cases are represented in Table 1.

**Table 1:** Results for the ETT and SGAD groups.

ETT Group					SGAD Group				
Case	ID (mm)	Weight (kg)	Bevel orientation	CT-scan slice thickness (mm)	Case	Size	Weight (kg)	Grade glottis closure	CT-scan slice thickness (mm)
1	4.5	5.2	Ventral wall	1	1	C5	5	0	0.75
2	4	5.2	Straight	1.5	2	C4	4.5	0	0.75
3	4.5	6.9	Ventral wall	0.75	3	C3	3.8	2	1
4	2.5	1.5	Ventral wall	0.75	4	C4	4.8	0	0.75
5	3.5	5.2	Ventral wall	1.5	5	C5	4.8	2	0.75
6	3.5	3	Lateral wall	1.5	6	C4	3.5	1	1.5
7	4	6.5	Ventral wall	1	7	C2	2.8	0	1.5
8	4	6.5	Dorsal wall	1.5	8	C2	2.5	1	1
9	4	5.6	Ventral wall	1	9	C6	6.5	2	1
10	3	4.4	Ventral wall	1	10	C4	3.8	2	0.75

For the ETT group, ID is the internal diameter of the ETT tube (mm), the weight of the patient in kg, the bevel orientation of the ETT related to the tracheal wall, and the slice thickness (mm) of the CT scan. For the SGAD group, the size of the device (C2 corresponds to the smallest size and C6 corresponds to the largest size), the weight of the patient in kg, grade of glottis closure (grade 0 = open glottis; grade 1 = glottis partially closed; and grade 2 = closed glottis) and slice thickness (mm) of the CT scan.

ETT, endotracheal tube; SGAD, supraglottic airway device; CT, computed tomography; ID, internal diameter.

The all bevels were, located at the level of the thoracic inlet. All ETTs included were PVC tubes with inflated cuff and had Murphy’s eye. No complete occlusion of the bevel’s tube or Murphy’s eye was observed. The tubes with a smaller diameter (ID 2.5–3 mm in cases #4 and #10, respectively) did not show higher values of velocity, pressure, and general resistance, but there was higher regional resistance in region A for case #4. Cases #1 and #3 had the largest diameter of ETT (ID = 4.5 mm). Case #1 showed the lowest general Δp, regional (A), and general resistance.

**Figure 5:**
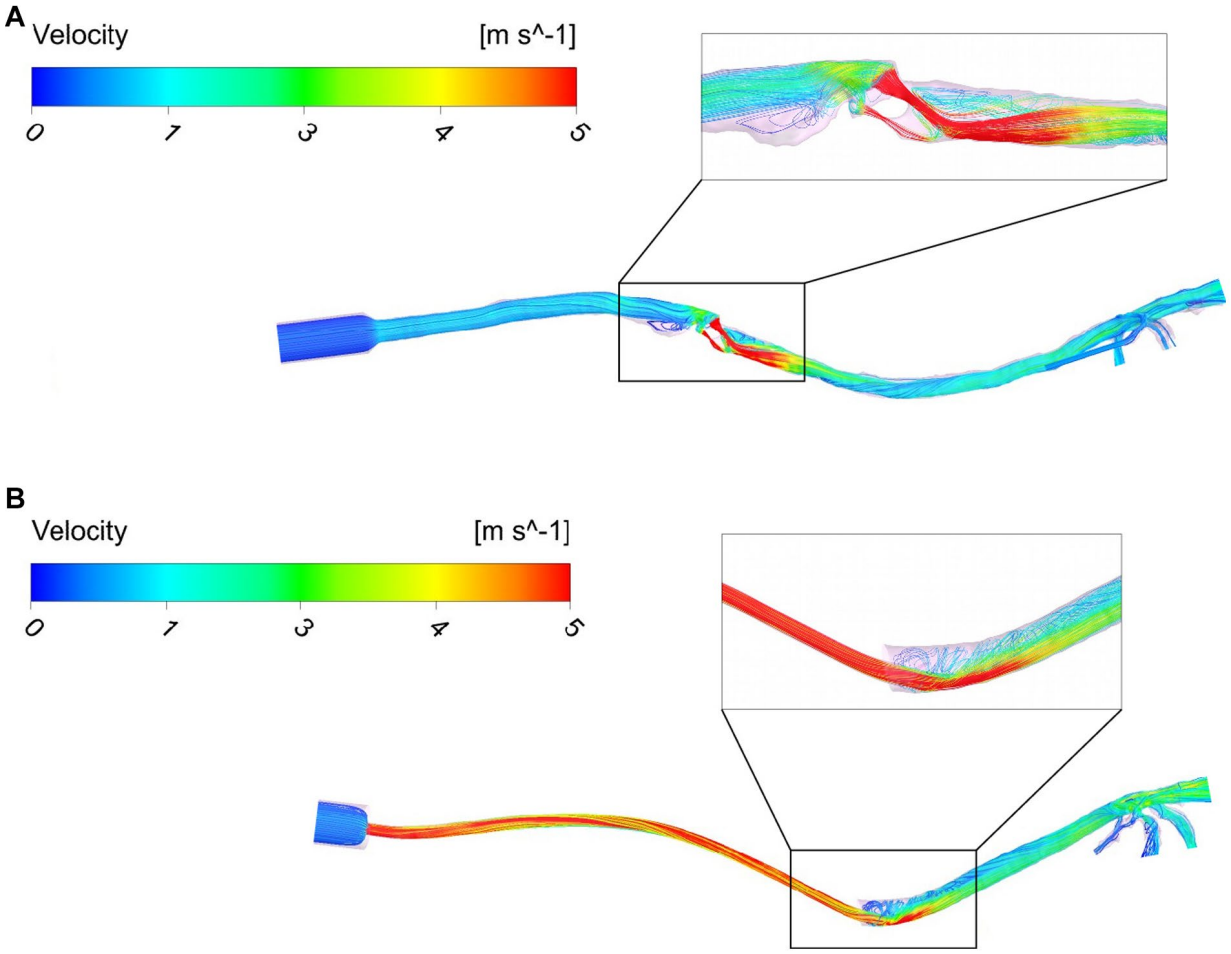
Flow maps using streamlines and colored with the velocity intensity (m/s) for a SGAD case (Case #5) **(A)** and an ETT case (Case #4) **(B)**. ETT: endotracheal tube; SGAD: supraglottic airway device. Flow streamlines represent the flow direction depicted with the intensity of the velocity (red = high velocity, dark blue = low velocity) at stationary peak inspiratory flow.

The flow maps depict the flow streamlines that represent the structure of the flow colored with the intensity of the velocity (red = high velocity, dark blue = low velocity) in m/s (see Figure 5). The flow streamlines of an ETT and a SGAD model (case #4 and case #5, respectively) are represented in Figure 5 at steady peak inspiratory flow. The rest of the cases can be found in the Suplemmentary material (Supplementary Figures 1, 2). In the ETT cases, the location with the highest velocities was the bevel of the tube contacting the wall of the trachea (9/10). In this region, the most pronounced flow recirculation was found. In all cases, a change in the velocity was seen in region A, where the diameter of the tube decreases with respect to the diameter of the tube connection to the breathing circuit. The velocities were higher as the diameter of the connection decreased. Mild outflow with minimal recirculation through Murphy’s eye was observed in all cases. Case #4 showed the maximum velocity (10.96 m/s). Only in one case (case #2) the bevel was aligned to the tracheal lumen. In the SGAD cases, the point with the highest velocities and flow recirculation lines were at the rima glottidis and caudal to it, consistent with CT image findings. In 6 cases, the glottis was partially (*n* = 2) or completely closed (*n* = 4). Cases #3, #5, #9, and #10 of the SGAD, classified as grade 2, showed the highest velocities (22.4 m/s, 21.1 m/s, 17.4 m/s, and 10.8 m/s, respectively). The median, maximum and minimum values of maximum velocity for the ETT and the SGAD groups are represented in Table 2. No statistically significant differences were observed between devices (*p* = 1.00).

**Table 2:** Results of each measured parameter (Ansys CFX software) represented by median [minimum value – maximum value] and the statistical significance represented by *p*-value (values of *p* < 0.05 were considered statistically significant).

Measured parameters	ETT group	SGAD group	*p*-value
Maximal velocity (m/s)	7.48 [4.01–10.96]	6.37 [3.32–22.4]	1.00
Maximal pressure (cmH_2_O)	1.07 [0.39–2.19]	0.39 [0.12–2.09]	0.09
Minimal pressure (cmH_2_O)	−0.01 [−0.04–0]	−0.07 [−2.32–0]	0.015
General Δp (cmH_2_O)	1.09 [0.4–2.19]	0.46 [0.13–4.37]	0.48
Region A Δp (cmH_2_O)	0.19 [0.07–0.46]	0.02 [0.01–0.05]	<0.001
Region B Δp (cmH_2_O)	0.02 [0.001–0.1]	0.23 [0.02–2.05]	0.001
Region C Δp (cmH_2_O)	0.95 [0.34–2.07]	0.33 [0.1–2.12]	0.63
Resistance A (cmH_2_O/L/min)	0.08 [0.03–0.2]	0.009 [0.005–0.23]	<0.001
Resistance B (cmH_2_O/L/min)	0.009 [0.0003–0.04]	0.09 [0.01–0.85]	0.001
Resistance C (cmH_2_O/L/min)	0.4 [0.15–0.86]	0.14 [0.04–0.88]	0.63
General resistance (cmH_2_O/L/min)	0.45 [0.16–0.91]	0.19 [0.05–1.82]	0.48

ETT, endotracheal tube; SGAD, supraglottic airway device; Δp, pressure gradient. Region A: consists of an area between two sections, localized where the proximal portion connects the device to the breathing circuit and the distal region where the device’s diameter decreases. Region B: consists of the area around the glottis in the SGAD group and, in the ETT group, consists in the point where the bevel contacts the tracheal wall. Region C: consists of the entire device.

**Figure 6:**
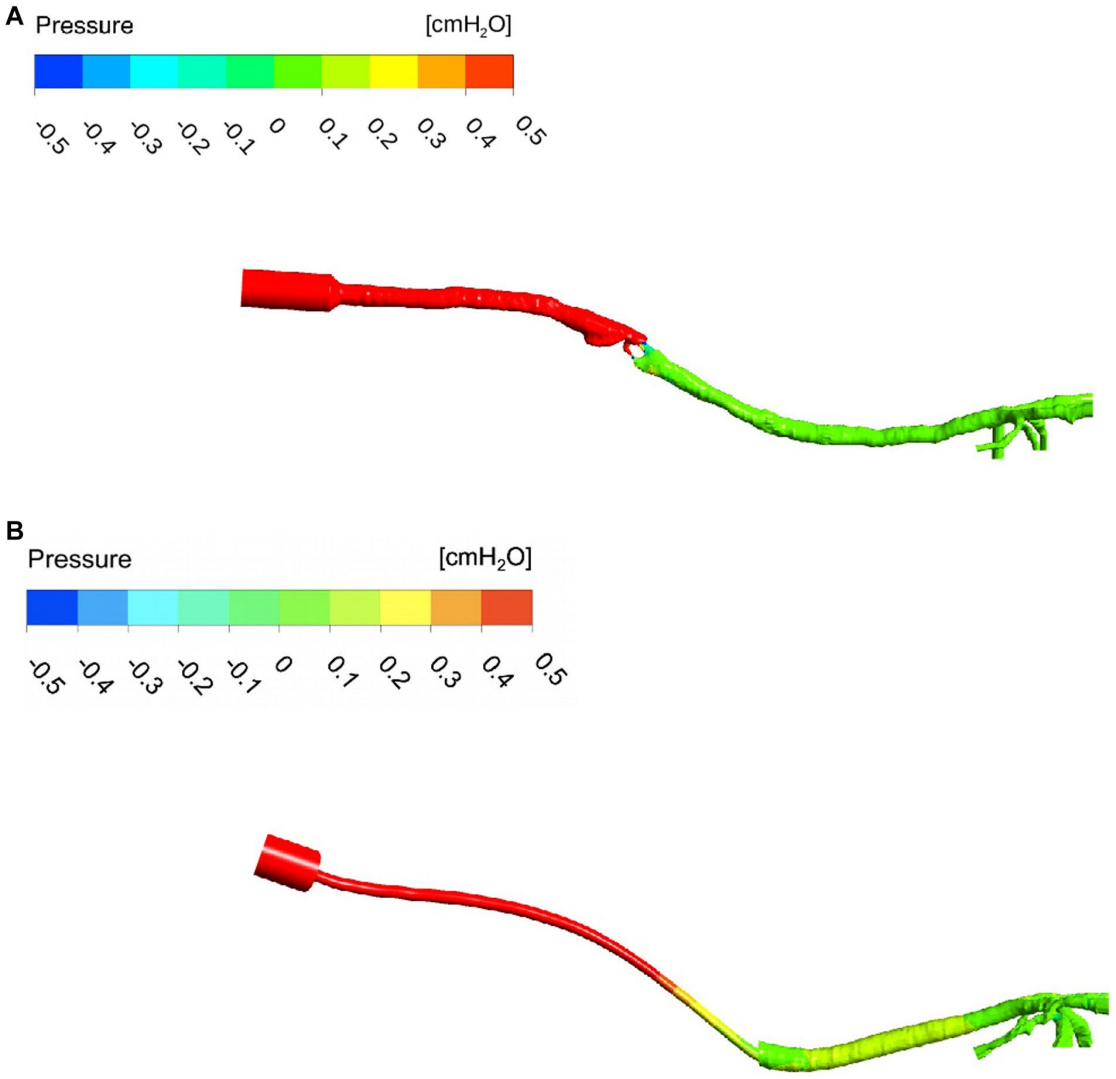
Pressure map of a SGAD (Case #5) **(A)** and an ETT (Case #4) **(B)**. The pressure gradient is staggered, from higher values (cmH_2_O) at the inlet to lower values at the outlets (bronchi). ETT: endotracheal tube; SGAD: supraglottic airway device; pressure values in cmH_2_O. The intensity of the pressure is represented at stationary peak inspiratory flow (red = high pressure, dark blue = low pressure).

The pressure maps of the device, trachea, and bronchi are represented for two examples of ETT and SGAD cases (case #4 and #5, respectively) in Figure 6 (negative and positive values represent increase and decrease with respect to the atmospheric pressure). The Δp was more staggered in the ETT case compared with the marked pressure change visible in the SGAD case. The rest of the pressure maps can be found in the Suplemmentary material (Supplementary Figures 3, 4). Higher pressures were observed cranial to the glottis in all SGAD cases, especially in cases #3, #5, #9, and #10. Case #3 of the SGAD had the highest maximum pressure value (2.09 cmH_2_O), and case #5 had the lowest minimum pressure value (−2.32 cmH_2_O). Both were classified as grade 2 glottic closures. The maximum pressures did not show statistically significant differences (*p* = 0.09), while the minimum pressures were statistically significant (*p* = 0.015) between both devices.

The median, maximum, and minimum values of general and regional Δp for the ETT and the SGAD groups (Table 2). The Δp in region A (*p* < 0.001) and B (*p* = 0.001) were statistically significant between groups but not in region C (*p* = 0.63). In region A, the Δp was higher in the ETT group, while in regions B and C, it was higher in the SGAD group. The maximum pressure value in region A was observed in case #4 of the ETT (ID = 2.5 mm). For regions B and C, the maximum pressure values (0.46 cmH_2_O) were for case #3 of the SGAD classified as grade 2 (2.05 cmH_2_O and 2.12 cmH_2_O, respectively).

The general resistance was not statistically significant between groups (*p* = 0.48). The maximum and minimum values were seen in the SGAD group. Higher values were observed in cases #3 and #5 (1.68 and 1.82 cmH_2_O/L/min, respectively), and the lowest value was observed in case #1 (0.05 cmH_2_O/L/min) and classified as grade 2 and 0, respectively. The resistance in region A was higher in the ETT group, and a statistically significant difference was found between devices (*p* < 0.001). The maximum value was observed in case #4 of the ETT (0.19 cmH_2_O/L/min). The resistance in region B was also statistically significant between groups (*p* = 0.001). In this region, higher values were obtained in the SGAD group, specifically in cases #3 and #5 (0.85 and 0.82 cmH_2_O/L/min, respectively). Case #10 (ID = 3 mm) showed the highest value in the ETT group in this region (0.04 cmH_2_O/L/min). The resistance in region C was not statistically significant (*p* = 0.63) between devices. High values were seen in the ETT group, but the highest resistance was observed in case #3 of the SGAD (0.88 cmH_2_O/L/min), classified as grade 2. The median, maximum, and minimum values of general and regional resistances for the ETT and the SGAD groups are represented in Table 2.

## 4. Discussion

This study computationally evaluated the performances of two devices for managing a cat’s airways: ETT and SGAD. We have found differences in Δp and resistances between the ETT and the SGAD groups at different device locations. The area of rima glottidis presented variable Δp and significantly higher values of resistance compared with the ETT group, as this device bypassed this region. However, in the case of the ETT, the region where the device connects to the breathing circuit has the highest resistance in this device. In any case, with these geometrical models and these flows, we could not see any differences in the general resistance in the device and airway models.

There is no consensus and poor knowledge in the veterinary field regarding whether different airway devices induce different flow patterns, Δp, and resistances. Physiological flows of 4–8 L/min (38) and 6 L/min (37) have been reported in awake cats. The inspiratory flow used in this study was smaller as we simulated the ventilation under general anesthesia (2.4 L/min). Consequently, the resistance, pressure, and velocity results are lower than other studies (34). Our results are based in CFD simulations. A comparison in the same patient with both devices was not performed. So it is unknown if changes in velocity, pressure and resistance are due to the devices alone or could be also caused by inter individual patient anatomical characteristics.

In our study, the ETT group’s highest velocity and pressure values were seen at the bevel. The ETTs used in this study are tubes for neonatal or pediatric human patients. Additionally, the intubation technique differs when compared with cats. The non-physiological ETT conformation for the cat resulted in the bevel impinging on the trachea’s wall in most cases, causing changes in the flow behavior. In humans, CFD studies have shown that the ETT induces swirls at the outlet of the straight ETT ending and is related to a jet flow effect (39). This effect may induce high flow rates in the trachea, creating a recirculation zone and the transition to turbulent flow (40). Lumb et al. pointed out that the position and direction of this jet can influence the distribution of the fresh gas flow in patients during general anesthesia with inhalant anesthetics (41). They described that the gas hits the carina or airway wall immediately, perturbing the laminar flow and generating recirculation near the large airways (41). In our study, multiple recirculatory streamlines and higher velocity in the ETT group were seen when the tube was closed to the tracheal wall, as described in humans (41). The same research group later studied the role of Murphy’s eye, finding that in a normally positioned ETT, most parts of the flow exited the tube through the bevel, and only a tiny fraction passed through Murphy’s eye (42). These results are similar to our findings.

In the SGAD cases, the largest number of recirculatory streamlines, higher velocity, and pressure values were seen at the glottis. Our CT scans showed the glottis wholly or partially closed in 6 of 10 SGAD cases. However, no relation was seen between SGAD size, glottis closure, and increased resistance. During this study, 3 of the 10 cases in the SGAD group needed a change in size of the device and/or orotracheal intubation resulting from obstruction problems during the anesthesia, none in the ETT group. We believe this complication was related to the sealing size, which is likely too big or not perfectly adaptable to all types of feline larynx size and morphology.

The area where the ETT is connected to the breathing circuit demonstrated a significant difference in Δp and resistance compared with the SGAD. This was expected as these connectors have a standardized 15 mm diameter, and the diameter change within the tube is abrupt. This connection is more abrupt for ETT than SGAD. However, we observed a lower Δp within each ETT model, as the flow can adapt to geometrical changes such as constriction and expansion (43). In any case, the diameter change is gradual in this region, so no recirculating streamlines were observed, although there was an increased velocity as the diameter decreased. In contrast, in the SGADs, the pressure maps clearly showed an abrupt Δp across the rima glottidis. Marków et al. reported that, in humans, the resistance to flow depends on the area of the rima glottidis opening, and it is independent of its shape (33). Our findings have partially relation with this study because cases with complete or partially occluded rima glottidis showed high velocities and Δp. In neonatal and pediatric human medicine, an association between ETT diameter and resistance has been reported (44). When the diameter of the ETT decreases, the resistance increases with significantly more work during breathing (44). However, in this study, the cases with smaller diameters of ETT did not show the highest values and no clear association between ETT size and the cat’s weight was seen.

We found similar values of resistances in the device itself and the whole model. In the ETT cases, the resistances in the device were higher than in the SGAD, likely because of the small diameter of the tube compared with the tracheal diameter. General resistance values of the SGAD group were twice as high as the ETT cases, but no statistical significance was observed. Moreover, the SGAD general resistances were uneven compared with the ETT cases, probably because of the regular diameter of the ETT. In the SGAD, patient anatomy and interindividual morphologic variability of the cats, especially at the laryngeal region, play an essential role in the studied flow parameters. This aspect may explain the heterogeneity of the data presented in this group. The general resistance values were dependent on the degree of rima glottidis closure, but not with the ETT connection system. The airway resistance falls with increasing airway bifurcations, and multiple factors play a role, including flow velocity, airway diameter, lung volume, and flow type (laminar or turbulent) (45).

This study has some limitations. Firstly, because of its retrospective and clinical nature, the CT studies have different slice thicknesses, and some were obtained for different regions (head, neck, and thorax). Secondly, intubation was carried out by all the anesthesia staff, interns, and students. The size of the ETT in some cases was smaller than expected for the size of the trachea or the cat’s weight. Another limitation is that the model includes the upper airways till the lobar bronchi so that the influence of the lower airways and alveoli was not evaluated. Further studies comparing healthy and diseased animals would be necessary to analyze velocity, pressure, or resistance changes. Finally, a patient-specific flow would provide precise information for accurate simulations and corresponding pressure and flow patterns. However, a comparison between two groups would be more difficult to be conducted, and ultimately that was the objective of this study. Studies comparing different flow parameters are required to relate resistance, velocity, and pressure values between different peaks in inspiratory flows and comparing *in silico* and *in vivo* data of the same patient using both devices.

In conclusion, we have proposed a CT-based CFD study for evaluating changes in airway velocity, pressure, and resistance between two airway management devices under general anesthesia. Their use in this study has showed a differences in resistance, pressure, and velocity at the ETT connector and outflow section and across the rima glottidis in the SGAD. So, selection of the correct device size is necessary to avoid airway obstructions or changes in the upper airway flow. This non-invasive technique can provide physiological and clinical information, as in human studies, but further investigations are needed to evaluate the specific application in veterinary medicine.

## Data availability statement

The original contributions presented in the study are included in the article/Supplementary material, further inquiries can be directed to the corresponding author.

## Ethics statement

The animal studies were approved by the Ethical committee of the Catholic University of Valencia (CEEAUCV2101). The studies were conducted in accordance with the local legislation and institutional requirements. Written informed consent was obtained from the owners for the participation of their animals in this study.

## Author contributions

CZ-P: data curation, formal analysis, investigation, resources, visualization, and writing – original draft. MM: conceptualization, formal analysis, investigation, methodology, project administration, resources, supervision, validation, visualization, and writing – review and editing. RF-P: conceptualization, data curation, formal analysis, investigation, methodology, project administration, supervision, validation, visualization, and writing – review and editing. All authors contributed to the article and approved the submitted version.

## Funding

MM and RF-P are supported by the grants PID2021-125731OB-C31 and PID2021-125731OB-C33, respectively, which are financed by the Spanish Ministry of Science and Innovation MCIN/AEI/10.13039/501100011033/ and FEDER (“A way to build Europe”).

## Conflict of interest

The authors declare that the research was conducted without any commercial or financial relationships that could be construed as a potential conflict of interest.

## Publisher’s note

All claims expressed in this article are solely those of the authors and do not necessarily represent those of their affiliated organizations, or those of the publisher, the editors and the reviewers. Any product that may be evaluated in this article, or claim that may be made by its manufacturer, is not guaranteed or endorsed by the publisher.
