# Supplementary material for: Computational fluid dynamics comparison of the upper airway velocity, pressure, and resistance in cats using an endotracheal tube or a supraglottic airway device
Source: Front Vet Sci. 2023 Sep 25;10:1183223. doi: 10.3389/fvets.2023.1183223 (PMC10561303; doi:10.3389/fvets.2023.1183223)
Supplement: Supplementary file 1 [file Data_Sheet_1.pdf]

Supplementary Material

Computational fluid dynamics comparison of the upper airway velocity, pressure, and resistance in cats using an endotracheal tube or supraglottic airway device

Carla Zamora-Perarnau*, Mauro Malvè, Rocío Fernández-Parra

*** Correspondence:** Carla Zamora-Perarnau carla.zamora@ucv.es

1 Supplementary Text: physics

Flow in the airways can be laminar or turbulent. Laminar flow can be represented through concentrically arranged cylinders with different flow rates and disposed in a telescopic organization. The closest cylinder to the wall of the tube has the slowest velocity due to the friction between air and wall. On the contrary, the cylinder located at the centre of the tube, i.e. tube axis, has the highest velocity(32).

When the tube wall can be considered rigid, the Poiseuille's Law that can be expressed through the following equation governs the airflow:

$$R = \frac{8\,\mu\, l}{\pi r^4}$$

Where μ is the dynamic viscosity and r and l are the tube radius and length respectively. The physical meaning of the Poiseuille's Law is the proportionality between pressure difference Δp and flow ϕ through the resistance R ($\Delta p \propto R\ \phi$).

Flow changes from laminar to turbulent, passing through an intermediate regime called transition, depending on a dimensionless number called Reynolds number and indicated with *Re*. This number is equal to the ratio between inertial and viscosity forces and can be written as:

$$Re = \frac{\rho\, v\, D}{\mu}$$

Where ρ is the density of the fluid, μ its dynamic viscosity, v its velocity and D the tube diameter.

During turbulent flow (Re > 2000), the relationship among the pressure difference, flow and resistance changes with respect to that obtained for laminar flow. The resistance is influenced more by the density than the viscosity, as the pressure difference is proportional to the square of the flow ($\Delta p \propto R\ \phi^2$). For this reason, to generate the same airflow is necessary much greater pressure difference. The organization of concentrically arranged tubes may be replaced by chaotic vortices of many sizes and intensities which interact with each other (phenomenon called recirculation) promoting consequently an increase of drag due to friction effects(32).

In the airflow, turbulent flow develops in large tubes with high airflow velocity and elevated gas density (mainly at the trachea and main bronchi). On the contrary, laminar flow occurs in small airways.

Furthermore, airflow can be steady or unsteady depending if the properties of the fluid (density, viscosity, velocity and pressures) change with the time or not. Generally, the flow in the airways is unsteady (or non stationary). However, in several situations it can be approximated as steady (or stationary), considering a specific time instant of the breathing cycle(35,36).

For solving the airflow in the cat airways in the presence of the devices, the Reynolds averaged Navier-Stokes equations were used. It is about the Navier-Stokes equations, that predict the three-dimensional flow in complex geometries, written for turbulent flow such the present cases. In these equations, the instantaneous variables are decomposed into their time-averaged and fluctuating quantities to take into account the turbulence. The resultant equations are solved iteratively by the commercial software used in this study, and can be written as:

$$\nabla \cdot \bar{\boldsymbol{v}} = \mathbf{0}$$

$$\frac{d\bar{\boldsymbol{v}}}{dt} + \nabla\,(\bar{\boldsymbol{v}} \times \bar{\boldsymbol{v}}) = -\frac{1}{\rho}\,\nabla p' + \nabla \cdot (\upsilon_{eff}(\nabla\bar{\boldsymbol{v}} + (\nabla\bar{\boldsymbol{v}})^T))$$

Where *d/dt* is the time derivative of the averaged fluid velocity vector $\bar{\boldsymbol{v}}$, ∇ is the mathematic differential operator, superscript ^{T} indicates that the gradient of the averaged velocity vector $\nabla\bar{\boldsymbol{v}}$ is transposed, ρ is the gas density, p' is modified pressure, υ_{eff} is the effective kinematic viscosity ($\upsilon_{eff} = \upsilon + \upsilon_t$, where υ is gas kinematic viscosity and υ_t is turbulence kinematic viscosity). Between the kinematic and the turbulent kinematic viscosities exists the relation $\upsilon_t = k / \omega$, where k represents the turbulence kinetic energy, ω the rate of dissipation of the turbulence kinetic energy.

2 Supplementary Figures and Tables

2.1 Supplementary Tables

ETT	Grid size (millions)	Computational cost (minutes)
Case 1	4.53	90
Case 2	3.59	129
Case 3	5.45	150
Case 4	3.27	66
Case 5	4.16	149
Case 6	3.06	72
Case 7	4.30	103
Case 8	4.55	161
Case 9	3.04	80
Case 10	3.58	140
**SGAD**		
Case 1	3.29	270
Case 2	2.78	104
Case 3	2.68	114
Case 4	3.35	64
Case 5	2.74	203
Case 6	2.60	90
Case 7	2.37	41
Case 8	2.07	51
Case 9	2.40	85
Case 10	2.63	223

Supplementary Table 1: Numerical discretization and computational cost of all ETT and SGAD models. ETT: endotracheal tube; SGAD: supraglottic airway device.

2.2 Supplementary Figures

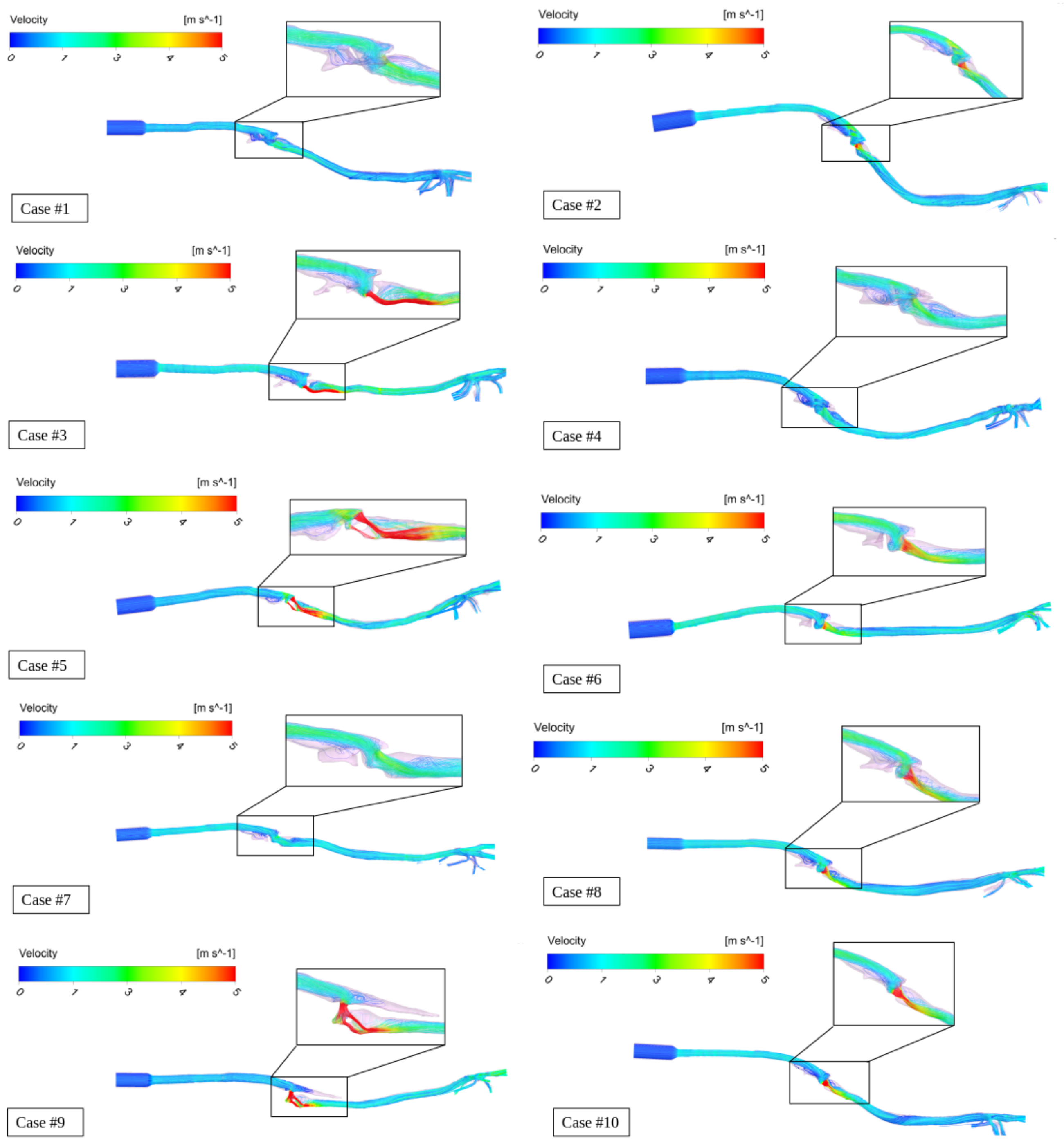

Supplementary Figure 1. Flow maps of all supraglottic airway device (SGAD) cases using streamlines and colored with the velocity intensity (m/s). Airflow streamlines represent the flow direction depicted with the intensity of the velocity (red = high velocity, dark blue = low velocity) at stationary peak inspiratory flow.

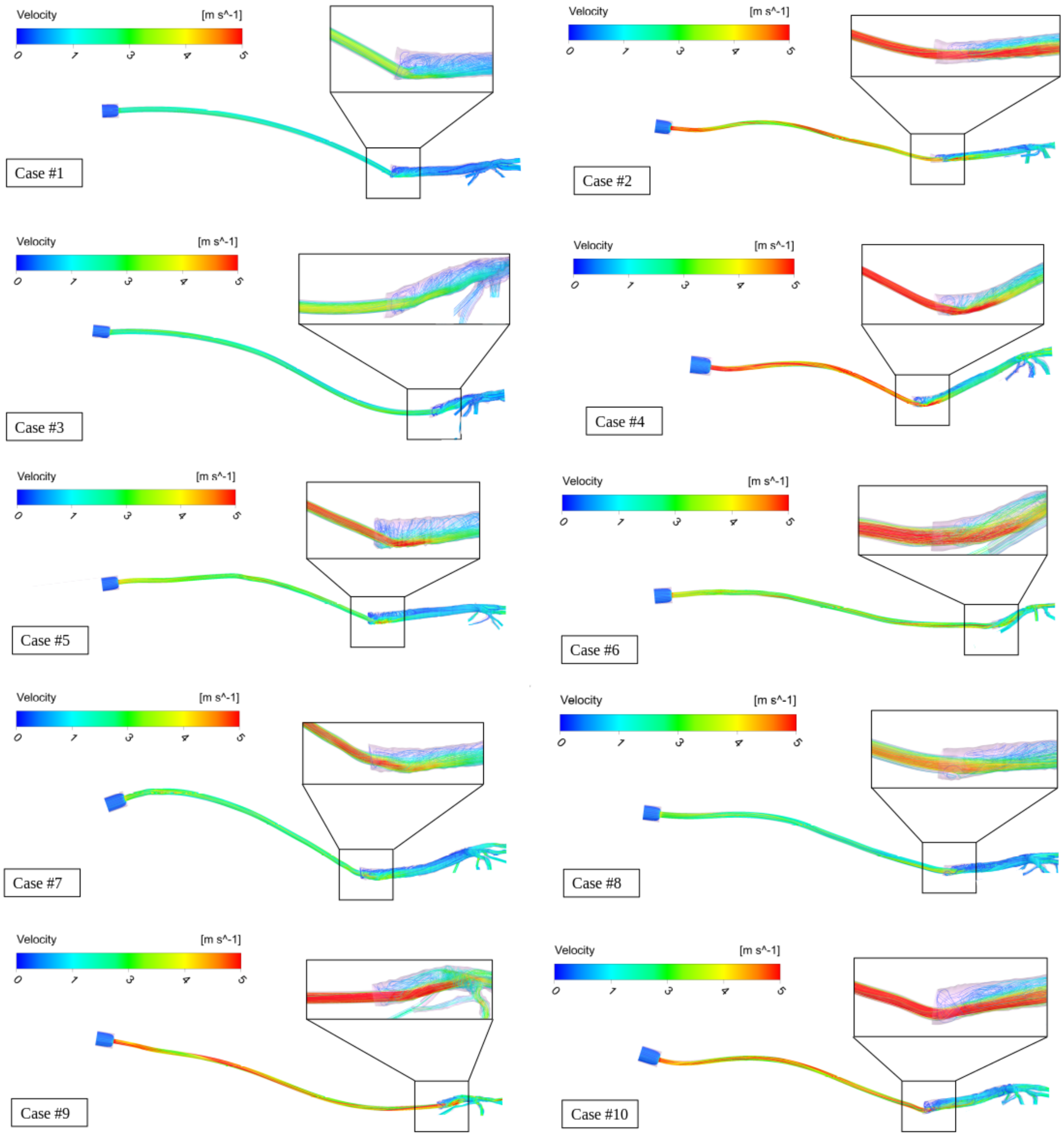

Supplementary Figure 2. Flow maps of all endotracheal tube (ETT) cases using streamlines and colored with the velocity intensity (m/s). Airflow streamlines represent the flow direction depicted with the intensity of the velocity (red = high velocity, dark blue = low velocity) at stationary peak inspiratory flow.

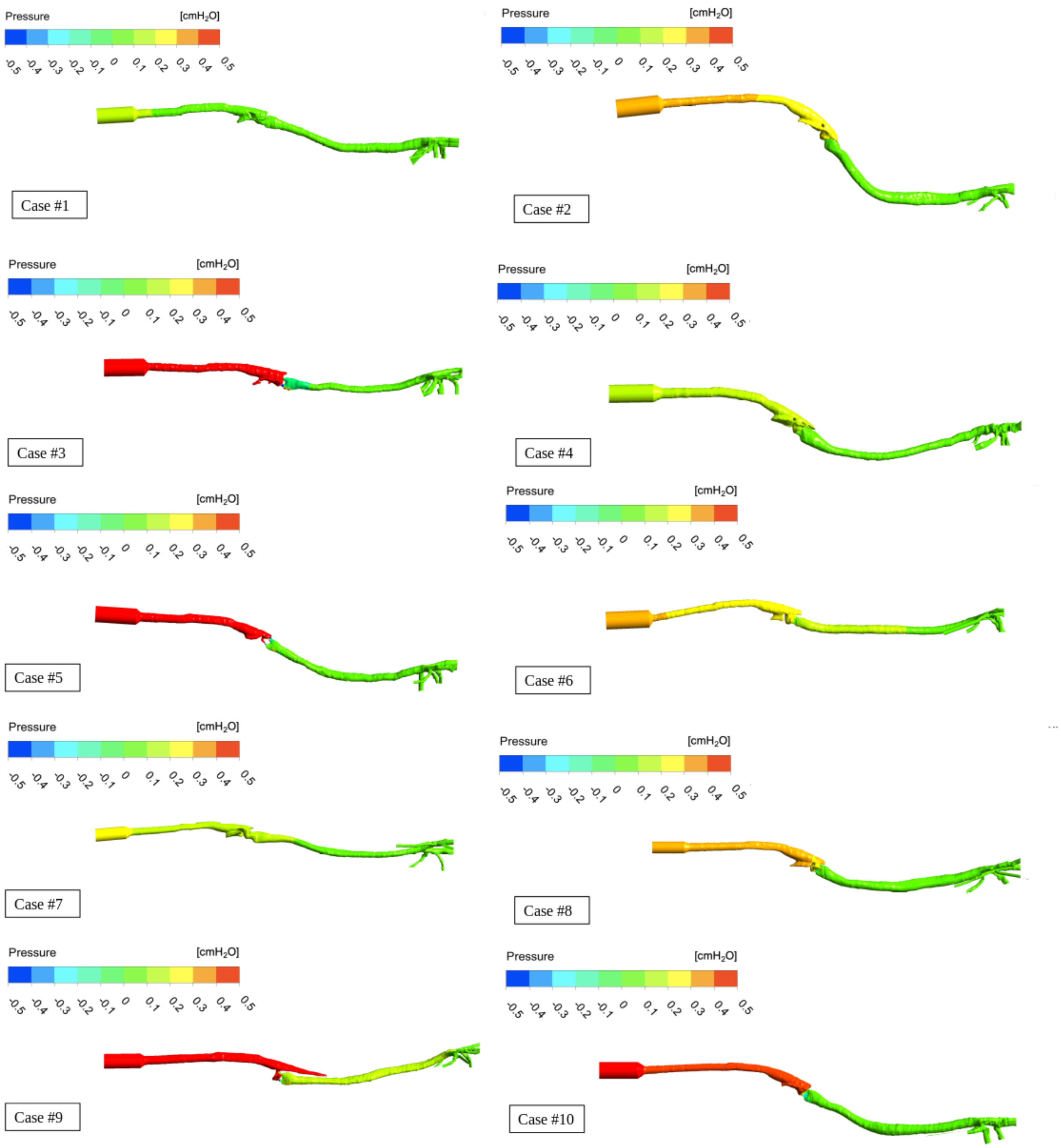

Supplementary Figure 3. Pressure maps of all supraglottic airway device (SGAD) cases. The intensity of the pressure is represented at stationary peak inspiratory flow (red = high pressure, dark blue = low pressure).

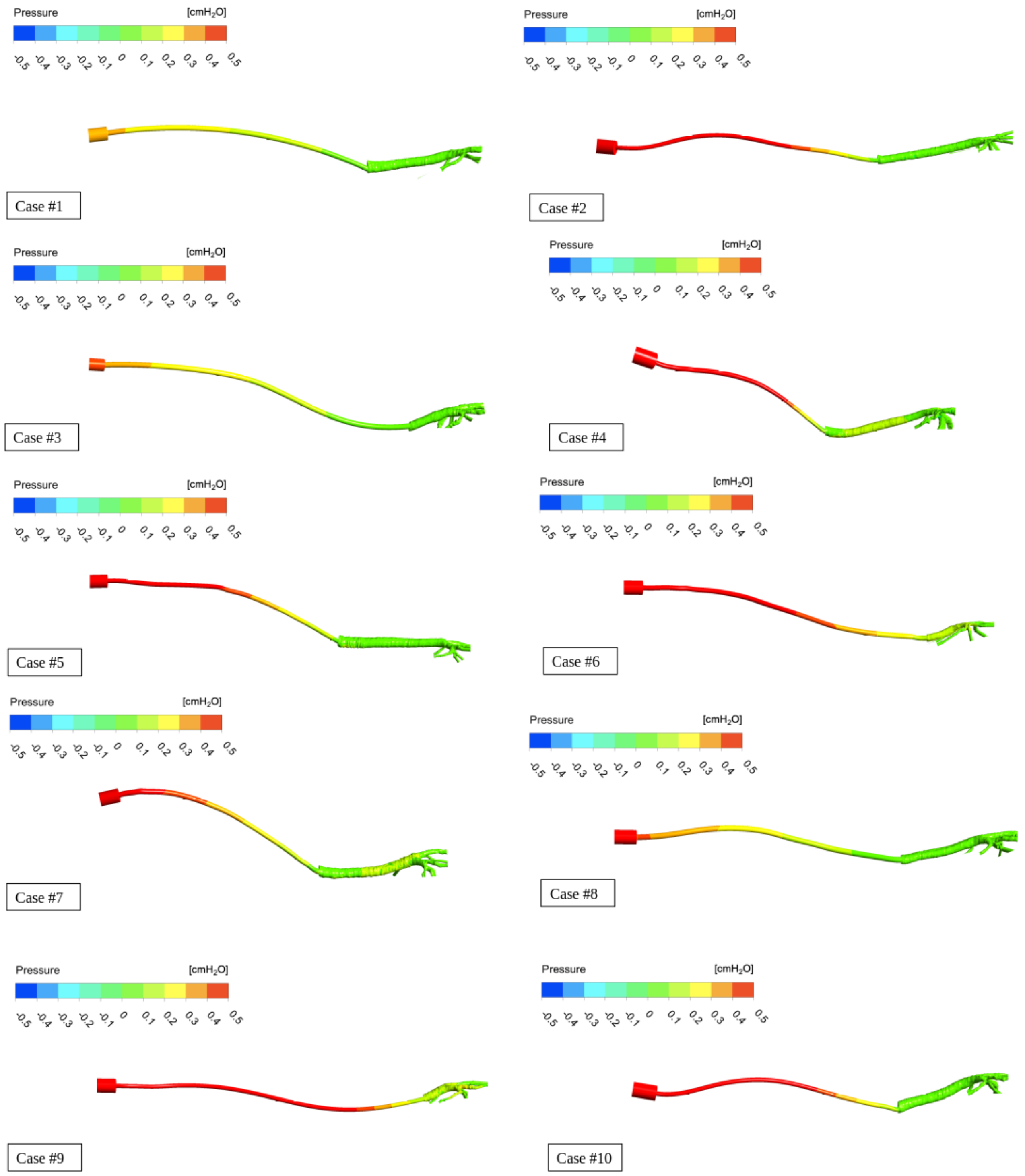

Supplementary Figure 4. Pressure maps of all endotracheal tube (ETT) cases. The intensity of the pressure is represented at stationary peak inspiratory flow (red = high pressure, dark blue = low pressure).
